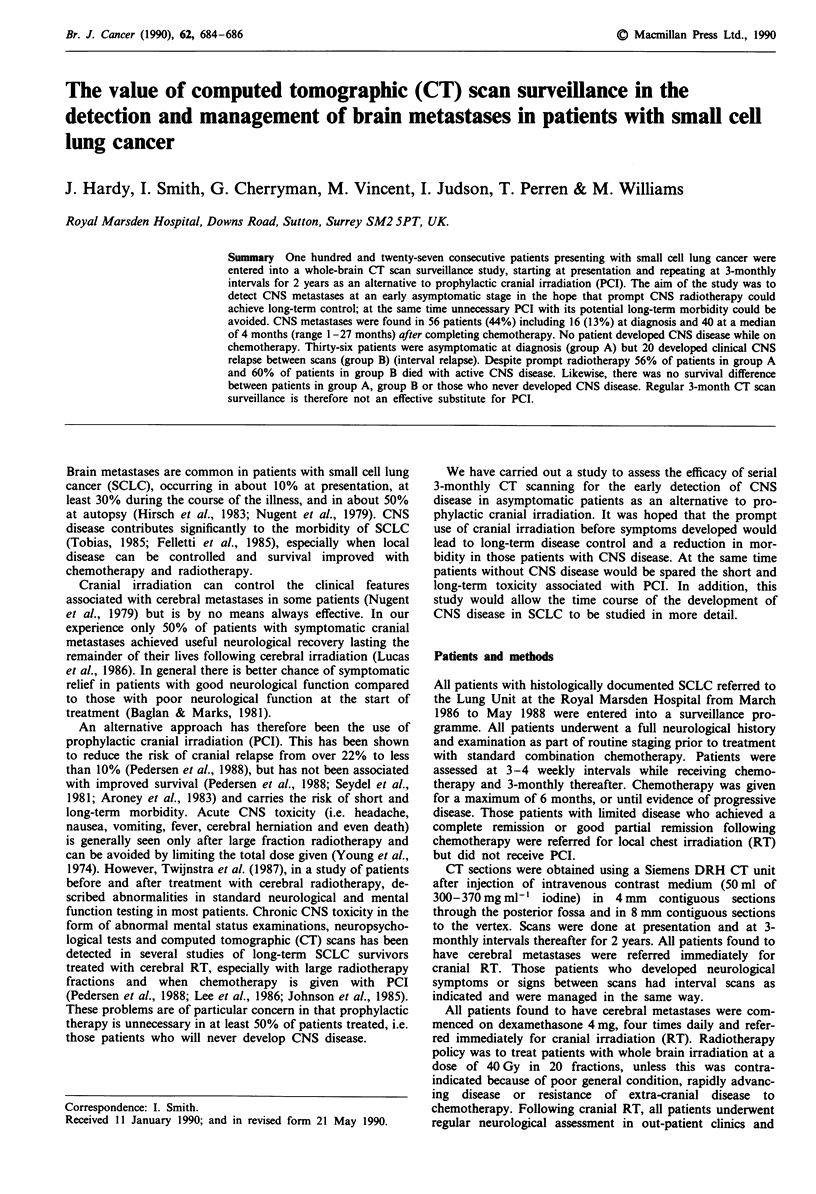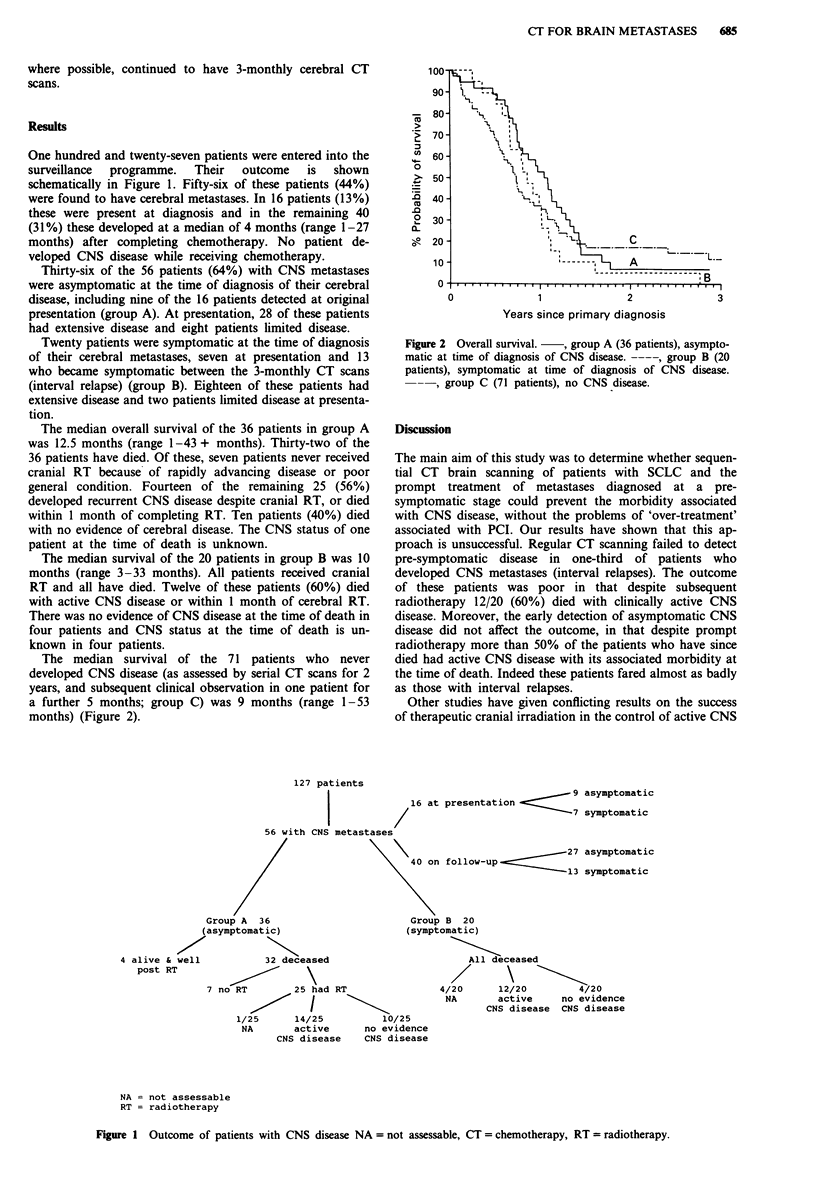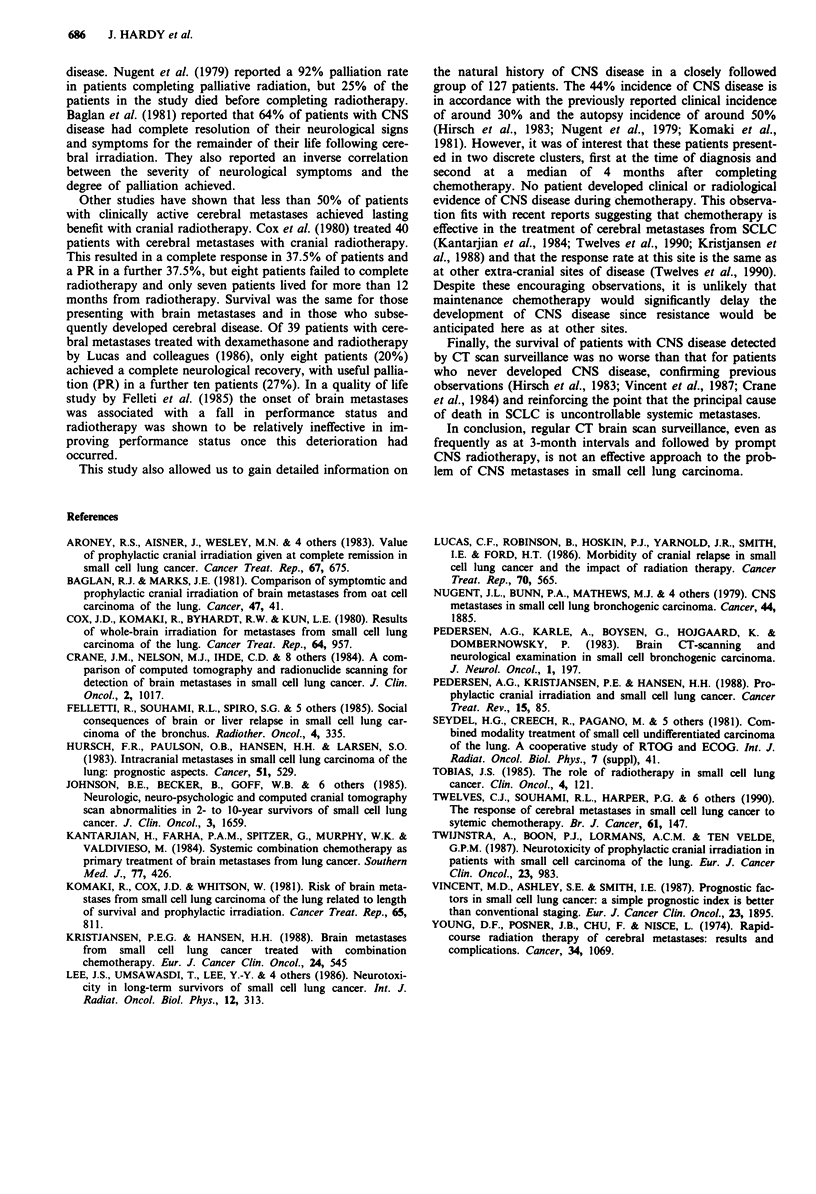# The value of computed tomographic (CT) scan surveillance in the detection and management of brain metastases in patients with small cell lung cancer.

**DOI:** 10.1038/bjc.1990.357

**Published:** 1990-10

**Authors:** J. Hardy, I. Smith, G. Cherryman, M. Vincent, I. Judson, T. Perren, M. Williams

**Affiliations:** Royal Marsden Hospital, Sutton, Surrey, UK.

## Abstract

One hundred and twenty-seven consecutive patients presenting with small cell lung cancer were entered into a whole-brain CT scan surveillance study, starting at presentation and repeating at 3-monthly intervals for 2 years as an alternative to prophylactic cranial irradiation (PCI). The aim of the study was to detect CNS metastases at an early asymptomatic stage in the hope that prompt CNS radiotherapy could achieve long-term control; at the same time unnecessary PCI with its potential long-term morbidity could be avoided. CNS metastases were found in 56 patients (44%) including 16 (13%) at diagnosis and 40 at a median of 4 months (range 1-27 months) after completing chemotherapy. No patient developed CNS disease while on chemotherapy. Thirty-six patients were asymptomatic at diagnosis (group A) but 20 developed clinical CNS relapse between scans (group B) (interval relapse). Despite prompt radiotherapy 56% of patients in group A and 60% of patients in group B died with active CNS disease. Likewise, there was no survival difference between patients in group A, group B or those who never developed CNS disease. Regular 3-month CT scan surveillance is therefore not an effective substitute for PCI.


*Br. J. Cancer* (1990), **62**, 684–686 © Macmillan Press Ltd., 1990

### The value of computed tomographic (CT) scan surveillance in the detection and management of brain metastases in patients with small cell lung cancer

J. Hardy, I. Smith, G. Cherryman, M. Vincent, I. Judson, T. Perren & M. Williams

*Royal Marsden Hospital, Downs Road, Sutton, Surrey SM2 5PT, UK.*

**Summary** One hundred and twenty-seven consecutive patients presenting with small cell lung cancer were entered into a whole-brain CT scan surveillance study, starting at presentation and repeating at 3-monthly intervals for 2 years as an alternative to prophylactic cranial irradiation (PCI). The aim of the study was to detect CNS metastases at an early asymptomatic stage in the hope that prompt CNS radiotherapy could achieve long-term control; at the same time unnecessary PCI with its potential long-term morbidity could be avoided. CNS metastases were found in 56 patients (44%) including 16 (13%) at diagnosis and 40 at a median of 4 months (range 1–27 months) *after* completing chemotherapy. No patient developed CNS disease while on chemotherapy. Thirty-six patients were asymptomatic at diagnosis (group A) but 20 developed clinical CNS relapse between scans (group B) (interval relapse). Despite prompt radiotherapy 56% of patients in group A and 60% of patients in group B died with active CNS disease. Likewise, there was no survival difference between patients in group A, group B or those who never developed CNS disease. Regular 3-month CT scan surveillance is therefore not an effective substitute for PCI.

Brain metastases are common in patients with small cell lung cancer (SCLC), occurring in about 10% at presentation, at least 30% during the course of the illness, and in about 50% at autopsy (Hirsch *et al.*, 1983; Nugent *et al.*, 1979). CNS disease contributes significantly to the morbidity of SCLC (Tobias, 1985; Felletti *et al.*, 1985), especially when local disease can be controlled and survival improved with chemotherapy and radiotherapy.

Cranial irradiation can control the clinical features associated with cerebral metastases in some patients (Nugent *et al.*, 1979) but is by no means always effective. In our experience only 50% of patients with symptomatic cranial metastases achieved useful neurological recovery lasting the remainder of their lives following cerebral irradiation (Lucas *et al.*, 1986). In general there is better chance of symptomatic relief in patients with good neurological function compared to those with poor neurological function at the start of treatment (Baglan & Marks, 1981).

An alternative approach has therefore been the use of prophylactic cranial irradiation (PCI). This has been shown to reduce the risk of cranial relapse from over 22% to less than 10% (Pedersen *et al.*, 1988), but has not been associated with improved survival (Pedersen *et al.*, 1988; Seydel *et al.*, 1981; Aroney *et al.*, 1983) and carries the risk of short and long-term morbidity. Acute CNS toxicity (i.e. headache, nausea, vomiting, fever, cerebral herniation and even death) is generally seen only after large fraction radiotherapy and can be avoided by limiting the total dose given (Young *et al.*, 1974). However, Twijnstra *et al.* (1987), in a study of patients before and after treatment with cerebral radiotherapy, described abnormalities in standard neurological and mental function testing in most patients. Chronic CNS toxicity in the form of abnormal mental status examinations, neuropsychological tests and computed tomographic (CT) scans has been detected in several studies of long-term SCLC survivors treated with cerebral RT, especially with large radiotherapy fractions and when chemotherapy is given with PCI (Pedersen *et al.*, 1988; Lee *et al.*, 1986; Johnson *et al.*, 1985). These problems are of particular concern in that prophylactic therapy is unnecessary in at least 50% of patients treated, i.e. those patients who will never develop CNS disease.

Correspondence: I. Smith.
Received 11 January 1990; and in revised form 21 May 1990.

We have carried out a study to assess the efficacy of serial 3-monthly CT scanning for the early detection of CNS disease in asymptomatic patients as an alternative to prophylactic cranial irradiation. It was hoped that the prompt use of cranial irradiation before symptoms developed would lead to long-term disease control and a reduction in morbidity in those patients with CNS disease. At the same time patients without CNS disease would be spared the short and long-term toxicity associated with PCI. In addition, this study would allow the time course of the development of CNS disease in SCLC to be studied in more detail.

### Patients and methods

All patients with histologically documented SCLC referred to the Lung Unit at the Royal Marsden Hospital from March 1986 to May 1988 were entered into a surveillance programme. All patients underwent a full neurological history and examination as part of routine staging prior to treatment with standard combination chemotherapy. Patients were assessed at 3–4 weekly intervals while receiving chemotherapy and 3-monthly thereafter. Chemotherapy was given for a maximum of 6 months, or until evidence of progressive disease. Those patients with limited disease who achieved a complete remission or good partial remission following chemotherapy were referred for local chest irradiation (RT) but did not receive PCI.

CT sections were obtained using a Siemens DRH CT unit after injection of intravenous contrast medium (50 ml of 300–370 mg ml$^{-1}$ iodine) in 4 mm contiguous sections through the posterior fossa and in 8 mm contiguous sections to the vertex. Scans were done at presentation and at 3-monthly intervals thereafter for 2 years. All patients found to have cerebral metastases were referred immediately for cranial RT. Those patients who developed neurological symptoms or signs between scans had interval scans as indicated and were managed in the same way.

All patients found to have cerebral metastases were commenced on dexamethasone 4 mg, four times daily and referred immediately for cranial irradiation (RT). Radiotherapy policy was to treat patients with whole brain irradiation at a dose of 40 Gy in 20 fractions, unless this was contraindicated because of poor general condition, rapidly advancing disease or resistance of extra-cranial disease to chemotherapy. Following cranial RT, all patients underwent regular neurological assessment in out-patient clinics and where possible, continued to have 3-monthly cerebral CT scans.

### Results

One hundred and twenty-seven patients were entered into the surveillance programme. Their outcome is shown schematically in Figure 1. Fifty-six of these patients (44%) were found to have cerebral metastases. In 16 patients (13%) these were present at diagnosis and in the remaining 40 (31%) these developed at a median of 4 months (range 1–27 months) after completing chemotherapy. No patient developed CNS disease while receiving chemotherapy.

Thirty-six of the 56 patients (64%) with CNS metastases were asymptomatic at the time of diagnosis of their cerebral disease, including nine of the 16 patients detected at original presentation (group A). At presentation, 28 of these patients had extensive disease and eight patients limited disease.

Twenty patients were symptomatic at the time of diagnosis of their cerebral metastases, seven at presentation and 13 who became symptomatic between the 3-monthly CT scans (interval relapse) (group B). Eighteen of these patients had extensive disease and two patients limited disease at presentation.

The median overall survival of the 36 patients in group A was 12.5 months (range 1–43 + months). Thirty-two of the 36 patients have died. Of these, seven patients never received cranial RT because of rapidly advancing disease or poor general condition. Fourteen of the remaining 25 (56%) developed recurrent CNS disease despite cranial RT, or died within 1 month of completing RT. Ten patients (40%) died with no evidence of cerebral disease. The CNS status of one patient at the time of death is unknown.

The median survival of the 20 patients in group B was 10 months (range 3–33 months). All patients received cranial RT and all have died. Twelve of these patients (60%) died with active CNS disease or within 1 month of cerebral RT. There was no evidence of CNS disease at the time of death in four patients and CNS status at the time of death is unknown in four patients.

The median survival of the 71 patients who never developed CNS disease (as assessed by serial CT scans for 2 years, and subsequent clinical observation in one patient for a further 5 months; group C) was 9 months (range 1–53 months) (Figure 2).

**Figure 2** Overall survival. ——, group A (36 patients), asymptomatic at time of diagnosis of CNS disease. ----, group B (20 patients), symptomatic at time of diagnosis of CNS disease. —·—, group C (71 patients), no CNS disease.

### Discussion

The main aim of this study was to determine whether sequential CT brain scanning of patients with SCLC and the prompt treatment of metastases diagnosed at a pre-symptomatic stage could prevent the morbidity associated with CNS disease, without the problems of 'over-treatment' associated with PCI. Our results have shown that this approach is unsuccessful. Regular CT scanning failed to detect pre-symptomatic disease in one-third of patients who developed CNS metastases (interval relapses). The outcome of these patients was poor in that despite subsequent radiotherapy 12/20 (60%) died with clinically active CNS disease. Moreover, the early detection of asymptomatic CNS disease did not affect the outcome, in that despite prompt radiotherapy more than 50% of the patients who have since died had active CNS disease with its associated morbidity at the time of death. Indeed these patients fared almost as badly as those with interval relapses.

Other studies have given conflicting results on the success of therapeutic cranial irradiation in the control of active CNS

**Figure 1** Outcome of patients with CNS disease NA = not assessable, CT = chemotherapy, RT = radiotherapy.

disease. Nugent et al. (1979) reported a 92% palliation rate in patients completing palliative radiation, but 25% of the patients in the study died before completing radiotherapy. Baglan et al. (1981) reported that 64% of patients with CNS disease had complete resolution of their neurological signs and symptoms for the remainder of their life following cerebral irradiation. They also reported an inverse correlation between the severity of neurological symptoms and the degree of palliation achieved.

Other studies have shown that less than 50% of patients with clinically active cerebral metastases achieved lasting benefit with cranial radiotherapy. Cox et al. (1980) treated 40 patients with cerebral metastases with cranial radiotherapy. This resulted in a complete response in 37.5% of patients and a PR in a further 37.5%, but eight patients failed to complete radiotherapy and only seven patients lived for more than 12 months from radiotherapy. Survival was the same for those presenting with brain metastases and in those who subsequently developed cerebral disease. Of 39 patients with cerebral metastases treated with dexamethasone and radiotherapy by Lucas and colleagues (1986), only eight patients (20%) achieved a complete neurological recovery, with useful palliation (PR) in a further ten patients (27%). In a quality of life study by Felleti et al. (1985) the onset of brain metastases was associated with a fall in performance status and radiotherapy was shown to be relatively ineffective in improving performance status once this deterioration had occurred.

This study also allowed us to gain detailed information on the natural history of CNS disease in a closely followed group of 127 patients. The 44% incidence of CNS disease is in accordance with the previously reported clinical incidence of around 30% and the autopsy incidence of around 50% (Hirsch et al., 1983; Nugent et al., 1979; Komaki et al., 1981). However, it was of interest that these patients presented in two discrete clusters, first at the time of diagnosis and second at a median of 4 months after completing chemotherapy. No patient developed clinical or radiological evidence of CNS disease during chemotherapy. This observation fits with recent reports suggesting that chemotherapy is effective in the treatment of cerebral metastases from SCLC (Kantarjian et al., 1984; Twelves et al., 1990; Kristjansen et al., 1988) and that the response rate at this site is the same as at other extra-cranial sites of disease (Twelves et al., 1990). Despite these encouraging observations, it is unlikely that maintenance chemotherapy would significantly delay the development of CNS disease since resistance would be anticipated here as at other sites.

Finally, the survival of patients with CNS disease detected by CT scan surveillance was no worse than that for patients who never developed CNS disease, confirming previous observations (Hirsch et al., 1983; Vincent et al., 1987; Crane et al., 1984) and reinforcing the point that the principal cause of death in SCLC is uncontrollable systemic metastases.

In conclusion, regular CT brain scan surveillance, even as frequently as at 3-month intervals and followed by prompt CNS radiotherapy, is not an effective approach to the problem of CNS metastases in small cell lung carcinoma.

### References

ARONEY, R.S., AISNER, J., WESLEY, M.N. & 4 others (1983). Value of prophylactic cranial irradiation given at complete remission in small cell lung cancer. *Cancer Treat. Rep.*, **67**, 675.

BAGLAN, R.J. & MARKS, J.E. (1981). Comparison of symptomtic and prophylactic cranial irradiation of brain metastases from oat cell carcinoma of the lung. *Cancer*, **47**, 41.

COX, J.D., KOMAKI, R., BYHARDT, R.W. & KUN, L.E. (1980). Results of whole-brain irradiation for metastases from small cell lung carcinoma of the lung. *Cancer Treat. Rep.*, **64**, 957.

CRANE, J.M., NELSON, M.J., IHDE, C.D. & 8 others (1984). A comparison of computed tomography and radionuclide scanning for detection of brain metastases in small cell lung cancer. *J. Clin. Oncol.*, **2**, 1017.

FELLETTI, R., SOUHAMI, R.L., SPIRO, S.G. & 5 others (1985). Social consequences of brain or liver relapse in small cell lung carcinoma of the bronchus. *Radiother. Oncol.*, **4**, 335.

HURSCH, F.R., PAULSON, O.B., HANSEN, H.H. & LARSEN, S.O. (1983). Intracranial metastases in small cell lung carcinoma of the lung: prognostic aspects. *Cancer*, **51**, 529.

JOHNSON, B.E., BECKER, B., GOFF, W.B. & 6 others (1985). Neurologic, neuro-psychologic and computed cranial tomography scan abnormalities in 2- to 10-year survivors of small cell lung cancer. *J. Clin. Oncol.*, **3**, 1659.

KANTARJIAN, H., FARHA, P.A.M., SPITZER, G., MURPHY, W.K. & VALDIVIESO, M. (1984). Systemic combination chemotherapy as primary treatment of brain metastases from lung cancer. *Southern Med. J.*, **77**, 426.

KOMAKI, R., COX, J.D. & WHITSON, W. (1981). Risk of brain metastases from small cell lung carcinoma of the lung related to length of survival and prophylactic irradiation. *Cancer Treat. Rep.*, **65**, 811.

KRISTJANSEN, P.E.G. & HANSEN, H.H. (1988). Brain metastases from small cell lung cancer treated with combination chemotherapy. *Eur. J. Cancer Clin. Oncol.*, **24**, 545

LEE, J.S., UMSAWASDI, T., LEE, Y.-Y. & 4 others (1986). Neurotoxicity in long-term survivors of small cell lung cancer. *Int. J. Radiat. Oncol. Biol. Phys.*, **12**, 313.

LUCAS, C.F., ROBINSON, B., HOSKIN, P.J., YARNOLD, J.R., SMITH, I.E. & FORD, H.T. (1986). Morbidity of cranial relapse in small cell lung cancer and the impact of radiation therapy. *Cancer Treat. Rep.*, **70**, 565.

NUGENT, J.L., BUNN, P.A., MATHEWS, M.J. & 4 others (1979). CNS metastases in small cell lung bronchogenic carcinoma. *Cancer*, **44**, 1885.

PEDERSEN, A.G., KARLE, A., BOYSEN, G., HOJGAARD, K. & DOMBERNOWSKY, P. (1983). Brain CT-scanning and neurological examination in small cell bronchogenic carcinoma. *J. Neurol. Oncol.*, **1**, 197.

PEDERSEN, A.G., KRISTJANSEN, P.E. & HANSEN, H.H. (1988). Prophylactic cranial irradiation and small cell lung cancer. *Cancer Treat. Rev.*, **15**, 85.

SEYDEL, H.G., CREECH, R., PAGANO, M. & 5 others (1981). Combined modality treatment of small cell undifferentiated carcinoma of the lung. A cooperative study of RTOG and ECOG. *Int. J. Radiat. Oncol. Biol. Phys.*, **7** (suppl), 41.

TOBIAS, J.S. (1985). The role of radiotherapy in small cell lung cancer. *Clin. Oncol.*, **4**, 121.

TWELVES, C.J., SOUHAMI, R.L., HARPER, P.G. & 6 others (1990). The response of cerebral metastases in small cell lung cancer to sytemic chemotherapy. *Br. J. Cancer*, **61**, 147.

TWIJNSTRA, A., BOON, P.J., LORMANS, A.C.M. & TEN VELDE, G.P.M. (1987). Neurotoxicity of prophylactic cranial irradiation in patients with small cell carcinoma of the lung. *Eur. J. Cancer Clin. Oncol.*, **23**, 983.

VINCENT, M.D., ASHLEY, S.E. & SMITH, I.E. (1987). Prognostic factors in small cell lung cancer: a simple prognostic index is better than conventional staging. *Eur. J. Cancer Clin. Oncol.*, **23**, 1895.

YOUNG, D.F., POSNER, J.B., CHU, F. & NISCE, L. (1974). Rapid-course radiation therapy of cerebral metastases: results and complications. *Cancer*, **34**, 1069.